# *CYP2C9* Pharmacogenetic Variants and Adverse Drug Reactions to Antiseizure Medications: An Exploratory Analysis in the Venezuelan Andes

**DOI:** 10.3390/ijms27198690

**Published:** 2026-09-29

**Authors:** Alexis Morales-Ortiz, Luis Abel Quiñones, Sol Betzabeth Rondón-Simancas, Gustavo Paredes, Andrea Avendaño, Zaida Gonzalez, Haydee Chávez, Juan J. Palomino-Jhong, María R. Bendezú, Jorge A. García, Elizabeth J. Melgar-Merino, Pompeyo A. Cuba-Garcia, Doris Laos-Anchante, Paulina Eliades Yarasca-Carlos, José Santiago Almeida-Galindo, Ricardo Pariona-Llanos, Felipe L. Ignacio-Cconchoy, Alvaro E. Maguiña-Ignacio, Berta Loja-Herrera, Richard Yaya-Araujo, Nelson M. Varela, Angel T. Alvarado

**Affiliations:** 1 Department of Toxicology and Pharmacology, Faculty of Pharmacy and Bioanalysis, University of Los Andes, Mérida 5101, Venezuela; toxicologoam@gmail.com (A.M.-O.); zaidagonzalez35@gmail.com (Z.G.); 2 Laboratory of Chemical Carcinogenesis and Pharmacogenetics, Department of Basic and Clinical Oncology, Faculty of Medicine, University of Chile, Santiago de Chile, Chile & Latin American Network for Implementation and Validation of Clinical Pharmacogenomics Guidelines (RELIVAF), Santiago 8350499, Chile; lquinone@med.uchile.cl; 3 Neurology Unit, Faculty of Medicine, University of Los Andes, Mérida 5101, Venezuela; drasolrondon@gmail.com (S.B.R.-S.); gustadopl@gmail.com (G.P.); 4 Medical Genetics Unit, Department of Paediatrics and Child Care, Faculty of Medicine, University of Los Andes, Mérida 5101, Venezuela; andrea.avendano1007@gmail.com; 5 Faculty of Pharmacy and Biochemistry, San Luis Gonzaga National University, Ica 11001, Peru; haydee.chavez@unica.edu.pe (H.C.); juan.palomino@unica.edu.pe (J.J.P.-J.); rocio.bendezu@unica.edu.pe (M.R.B.); jorge.garcia@unica.edu.pe (J.A.G.); elizabeth.melgar@unica.edu.pe (E.J.M.-M.); pompeyo.cuba@unica.edu.pe (P.A.C.-G.); doris.laos@unica.edu.pe (D.L.-A.); 6 Biological Sciences Faculty, San Luis Gonzaga National University, Ica 11001, Peru; eliades.yarasca@unica.edu.pe; 7 Faculty of Human Medicine, San Luis Gonzaga National University, Ica 11001, Peru; santiago.almeida@unica.edu.pe; 8 Biotechnology, Faculty of Environmental Engineering, University of Applied Sciences, Lima 15067, Peru; ricardo.pariona@upc.edu.pe; 9 Internal Medicine Department, Alberto Sabogal National Hospital, Sologuren, Callao 07000, Peru; fignacio@usil.edu.pe; 10 Centre for Research in Molecular Pharmacology and 4P Medicine, Vice-Rectorate for Research, San Ignacio de Loyola University, La Molina, Lima 15024, Peru; alvaro_erik_@hotmail.com (A.E.M.-I.); berta_loja@yahoo.com (B.L.-H.); richardyaya48@gmail.com (R.Y.-A.)

**Keywords:** *CYP2C9*, antiseizure medication, adverse drug reactions, pharmacogenetics, Venezuelan patients

## Abstract

Epilepsy is a chronic neurological disorder with a high global prevalence. In Venezuela, the limited availability of epidemiological and pharmacogenetic data represents an important public health challenge. Antiseizure medications (ASMs) are frequently associated with adverse drug reactions (ADRs), which may be influenced by genetic variability in drug-metabolising enzymes. This study evaluated the association between the CYP2C9*2 and CYP2C9*3 variants and the occurrence of ADRs in patients receiving anticonvulsant treatment in the Venezuelan Andes. An observational, analytical, cross-sectional study was conducted in 72 patients. Genomic DNA was extracted from venous blood, and *CYP2C9* genotypes were determined by real-time PCR using TaqMan^®^ probes. The frequencies of the CYP2C9*2 and CYP2C9*3 alleles were 0.15 and 0.06, respectively. The metaboliser phenotype was significantly associated with the presence of ADRs. The risk of ADRs was elevated in the intermediate/poor metaboliser group (IM/PM: OR = 17.45; 95%CI: 5.09–76.56; *p* < 0.001) and in patients on polypharmacy (OR = 15.03; 95%CI: 2.73–157.37; *p* = 0.001), although this latter estimate showed marked imprecision due to the width of its interval. Consistent with this, the ADR rate was higher in PM (100%; 3/3) and IM (84.2%; 16/19) than in normal group (20.9%; 9/43). In the monotherapy subgroup, the type of ASM showed no association with the occurrence of ADR (*p* = 0.741). Among normal metabolisers receiving polytherapy, regimens containing valproic acid were associated with the highest proportion of ADRs, with dual therapy including lamotrigine accounting for 28.57%. Although the overall analysis indicated differences in ADR occurrence according to polytherapy regimen (*p* = 0.043), the limited sample size restricts the strength of these findings. These results suggest that CYP2C9*2 and CYP2C9*3 variants may contribute to variability in the safety of anticonvulsant treatment among patients from the Venezuelan Andes by influencing CYP2C9 metaboliser phenotypes, and consequently, drug pharmacokinetics. Further studies in larger and more representative Venezuelan populations are warranted to validate these findings.

## 1. Introduction

Epilepsy is a neurological disorder characterised by recurrent, unprovoked seizures. Its aetiology may be genetic, either inherited or arising de novo from a mutation, or non-genetic, resulting from acquired lesions, structural abnormalities such as brain tumours, or causes of unknown origin [1,2]. It is estimated that more than 50 million people worldwide are affected by this condition, approximately 80% of whom live in emerging economies [1,3]. However, epidemiological data on epilepsy in Venezuela remain limited, representing a significant public health concern [4].

To control seizures, first-generation antiseizure medications (ASMs), including carbamazepine (CBZ), phenytoin (PHT), phenobarbital (PHB), and valproic acid (VPA), continue to be widely used, alongside second-generation agents such as lamotrigine (LTG), levetiracetam (LEV), and oxcarbazepine (OXC) [5]. These drugs generally have relatively narrow therapeutic indices, meaning that small variations in dosage or exposure may result in plasma concentrations below the minimum effective concentration, potentially contributing to inadequate seizure control and therapeutic failure, or above the minimum toxic concentration, increasing the risk of adverse drug reactions (ADRs) [6,7].

In addition to non-individualised dosing, several non-genetic factors may influence ASM metabolism and contribute to variability in plasma drug concentrations, including poor treatment adherence, impaired hepatic and/or renal function, age, body weight, and seizure type [8,9]. Genetic factors may also contribute to interindividual variability. Accordingly, polymorphisms in genes encoding enzymes involved in ASM metabolism have been investigated, including CYP2C9, CYP2C19, CYP3A4, UGT1A4, UGT2B7, and UGT2B15 [8]. In addition, the C3435T variant of the ABCB1 gene, which encodes an efflux transporter, has been investigated in relation to variability in ASM response [10].

Precision or personalised medicine aims to individualise drug therapy from the initiation of pharmacological treatment by incorporating relevant patient characteristics, including ethnicity, genetic ancestry or admixture, sex, lifestyle, and pharmacogenetic profile. The objective is to optimise drug exposure and maintain plasma concentrations within the therapeutic range while reducing the risk of treatment failure and toxicity [11]. In this context, the *CYP2C9* gene warrants particular attention because it encodes one of the major hepatic drug-metabolising enzymes. CYP2C9 is responsible for the metabolism of approximately 20% of clinically prescribed medicines and is particularly relevant for drugs with a narrow therapeutic index, for which even modest reductions in enzymatic activity may increase the risk of toxicity [12]. The CYP2C9 enzyme contributes to the metabolism of several first-generation ASMs, including PHT, PHB, and VPA [3].

Among the clinically relevant *CYP2C9* variants, CYP2C9*2 (rs1799853) and CYP2C9*3 (rs1057910) are particularly important. Both variants encode enzymes with reduced metabolic activity and have been associated with altered pharmacokinetics and an increased risk of toxicity for several drugs, including ASMs [13,14,15,16,17,18,19,20].

Figure 1 illustrates a schematic representation of chromosome 10, the structure of the *CYP2C9* gene, and the two allelic variants investigated in the present study.

Several ASMs are metabolised by cytochrome P450 enzymes, including CYP2C9. CBZ undergoes biotransformation mediated by CYP3A4, CYP3A5, CYP2C9, CYP2B6, and CYP3A7, generating metabolites such as carbamazepine-10,11-epoxide and carbamazepine catechol [21,22,23,24,25]. VPA is metabolised by CYP2C9, CYP2C19, CYP2B6, and CYP2A6, producing hydroxy– and ene– valproate metabolites [25,26]. Similarly, PHT is primarily metabolised by CYP2C9 and CYP2C19 to phenytoin-3′,4′-epoxide and its major metabolite, 5-(p-hydroxyphenyl)-5-phenylhydantoin (p-HPPH) [14,27]. PHB is also partially metabolised by CYP2C9, together with CYP2C19 and CYP2E1, to p-hydroxyphenobarbital [28].

CYP2C9 was selected because of its established role in the metabolism of several commonly used ASMs and the availability of clinically actionable pharmacogenetic guidelines [29]. The CYP2C9*2 and CYP2C9*3 alleles were selected because they are among the clinically most relevant variants affecting CYP2C9 activity and the metabolism of ASMs such as PHT and VPA, both of which are widely used in Venezuela. These reduced-function variants may result in decreased enzymatic activity, increased plasma drug concentrations, and a higher risk of toxicity under standard dosing regimens [30,31].

Despite the clinical relevance of CYP2C9 in the metabolism of several ASMs, pharmacogenetic studies in Venezuelan patients with epilepsy remain scarce. Consequently, the prevalence of clinically actionable *CYP2C9* phenotypes and their potential association with ADRs remains poorly characterised. Given this evidence gap, investigating *CYP2C9* variants with established clinical relevance represents a pragmatic approach to generating locally relevant pharmacogenetic evidence and supporting future implementation strategies in the region [32].

A review of the PubMed database identified limited evidence regarding *CYP2C9* allelic variation in Venezuelan patients with epilepsy. Accordingly, this study was designed to address this gap by: (i) determining the frequencies of *CYP2C9* alleles and genotypes among patients with epilepsy from the Venezuelan Andean region (Mérida State); (ii) evaluating the association between the CYP2C9*2 and CYP2C9*3 variants and ADRs to ASMs; and (iii) generating preliminary evidence regarding the potential clinical relevance of *CYP2C9* genetic variation in Venezuelan patients with epilepsy. Early identification of these polymorphisms may contribute to the identification of patients at increased risk of ADRs and may inform future pharmacogenetic approaches to antiseizure medication therapy [9].

The objective of this study was to characterise CYP2C9*2 and CYP2C9*3 allele and genotype frequencies, infer CYP2C9 metaboliser phenotypes, and evaluate their association with ADRs in patients receiving ASMs in the Venezuelan Andean region.

We hypothesised that carriers of the reduced-function *CYP2C9* alleles (*2 and *3), corresponding to intermediate and poor metaboliser phenotypes, would have a higher frequency of antiseizure medication-related ADRs than normal metabolisers (NM).

## 2. Results

### 2.1. Demographic, Clinical, and Medication Data

Analysis of the sociodemographic and anthropometric characteristics of the 72 patients with epilepsy revealed no statistically significant differences between men (*n* = 28; 38.9%) and women (*n* = 44; 61.1%) in terms of age (mean ± SD: 43.21 ± 18.85 vs. 46.45 ± 17.54 years, respectively; *p* = 0.429) or body weight (66.01 ± 14.46 vs. 60.79 ± 11.46 kg, respectively; *p* = 0.066) (Table 1). Monotherapy was the most frequently prescribed treatment regimen in both sexes (*n* = 65). CBZ and VPA were the most frequently prescribed ASMs, with comparable doses between female and male patients.

Polytherapy was prescribed to a relatively small proportion of patients (*n* = 7), with a similar distribution between sexes. Regarding seizure type and epilepsy control status, no statistically significant sex-related differences were identified. Likewise, no association was observed between sex and pharmacotherapy regimen (Fisher’s exact test, *p* = 1.000).

The similar distribution of demographic characteristics and treatment regimens between sexes reduces the potential for sex-related confounding in subsequent analyses of the association between the CYP2C9 metaboliser phenotype and the occurrence of ADRs.

### 2.2. CYP2C9 Allele and Genotype Frequencies

Genotype and allele frequencies were determined for the two selected *CYP2C9* single nucleotide polymorphisms (SNPs), rs1799853 (c.430C>T) and rs1057910 (c.1075A>C), which define the CYP2C9*2 and CYP2C9*3 alleles, respectively. Genotype frequencies for each variant were initially evaluated independently and are presented as marginal genotype frequencies in Table 2. For the CYP2C9*2-defining variant, the CYP2C9*1/*2 and CYP2C9*2/*2 genotypes were observed in 16.67% and 6.94% of patients, respectively. For the CYP2C9*3-defining variant, the CYP2C9*1/*3 genotype was observed in 11.00% of patients. No individuals carrying the CYP2C9*3/*3 genotype were identified.

The genotype frequencies of the CYP2C9*3-defining variant were consistent with the Hardy–Weinberg equilibrium (HWE; χ^2^ = 0.249, df = 1, *p* > 0.05). In contrast, the CYP2C9*2-defining variant showed a statistically significant deviation from the genotype proportions expected under HWE (χ^2^ = 9.134, df = 1, *p* < 0.05).

Subsequently, both loci were analysed jointly to infer *CYP2C9* diplotypes and predicted metaboliser phenotypes (Table 3). Accordingly, the apparent difference in the number of individuals classified as *1/*1 between Table 2 and Table 3 reflects the distinction between locus-specific marginal genotypes and inferred two-locus diplotypes, rather than a discrepancy in the underlying data.

Individuals heterozygous for both variants (rs1799853 CT and rs1057910 AC) were classified as CYP2C9*2/*3. This diplotype was inferred based on the established haplotype structure of *CYP2C9*, in which the variants defining the *2 and *3 alleles are generally located on distinct haplotypes and are rarely observed on the same chromosome [33]. Previous haplotype studies, including those based on phased genotypes from the 1000 Genomes Project, support a predominantly trans configuration of rs1799853-T and rs1057910-C [34,35]. However, because the genotyping method used in the present study did not directly determine the haplotype phase, the CYP2C9*2/*3 diplotype was considered inferred rather than directly phase determined.

CYP2C9 activity scores were calculated according to the Clinical Pharmacogenetics Implementation Consortium (CPIC) classification, assigning activity values of 1.0, 0.5, and 0 to the normal-function (*1), decreased-function (*2), and no-function (*3) alleles, respectively. The activity score was calculated as the sum of the allele-specific activity values and was subsequently used to assign predicted metaboliser phenotypes [13]. NM constituted the most common phenotype, accounting for 69.45% of individuals, followed by intermediate metabolisers (IM) at 26.38% and poor metabolisers (PM) at 4.17%. Phenotype assignment was based on the inferred *CYP2C9* diplotypes and their corresponding activity scores, in accordance with established pharmacogenetic guidelines.

### 2.3. Metaboliser Phenotype and ADRs

Table 4 presents the safety profile of ASMs used as monotherapy among the 65 patients receiving monotherapy, stratified according to CYP2C9 metaboliser phenotype. A statistically significant association was observed between metaboliser phenotype and the occurrence of ADRs (χ^2^ = 16.52; *p* 0.001). The frequency of ADRs was higher among IM (84.2%; 16/19) and PM (100%; 3/3) than among NM (20.9%; 9/43). In contrast, the occurrence of ADRs was not significantly associated with the type of ASM prescribed as monotherapy (χ^2^ test, *p* = 0.741).

The analysis of patients under ASM polytherapy regimens (*n* = 7) revealed an overall incidence of ADRs of 85.71% (*n* = 6; Table 5). VPA-based regimens accounted for the largest proportion of adverse events, with VPA + LTG biotherapy standing out (*n* = 2; 28.57% of the polytherapy sample). From a pharmacogenetic perspective, a complete absence of structural overlap was identified in this subgroup: 100% of the patients uniformly shared the normal metaboliser genotype (CYP2C9*1/*1). This genotypic homogeneity, together with the limited sample size, creates an intrinsic collinearity that prevents disentangling whether the risk of ADRs is determined by the pharmacogenomic profile or by pharmacological interactions (such as the well-known inhibitory effect of VPA on lamotrigine glucuronidation). Finally, the statistically significant difference detected in the overall analytical analysis (Fisher’s exact test, *p* = 0.043) refers strictly to the comparison of the ADR rate between the monotherapy group and the polytherapy group. This significance cannot be extrapolated to safety variations between the specific polytherapy sub-regimens, which were not subjected to intergroup comparisons due to the descriptive and limited nature of these data.

The initial multivariable model included five variables selected through expert judgement and clinical relevance: *CYP2C9* phenotype, ASM polytherapy, type of epilepsy, sex, and age. To ensure the mathematical stability of the regression and avoid overfitting resulting from the sample size, age and sex were excluded from the final model. Neither variable acted as a confounder and both showed a complete absence of association in the previous exploratory stages (e.g., sex versus polytherapy, Fisher’s exact test, *p* = 1.000), allowing a more parsimonious estimation of the critical predictors. To correct for bias due to diagnostic separation (monotone sparsity) and optimise the estimates in light of the small sample size, the final model was calculated using Firth’s penalised logistic regression. The confidence intervals (95% CIs) were estimated using the profile likelihood, and *p*-values were derived from the penalised likelihood ratio test. Prior to the interpretation of the coefficients, the statistical assumptions of the model were verified.

Multicollinearity: Its presence among the independent variables was ruled out, recording a maximum variance inflation factor (VIF) of 2.19.

Calibration: The Hosmer–Lemeshow goodness-of-fit test showed no evidence of lack of fit (χ^2^ = 0.67; df = 3; *p* = 0.880). For this analysis, the sample was partitioned into quintiles because tied predicted probabilities prevented the conventional division into deciles.

As observed in Table 6, the IM/PM phenotype (OR = 17.45; 95% CI: 5.09–76.56; *p* < 0.001) and polytherapy (OR = 15.03; 95% CI: 2.73–157.37; *p* = 0.001) were independently and statistically significantly associated with a higher risk of ADR. Although the direction and magnitude of the associations suggest strong clinical consistency, the notable width of the 95% CI reflects the statistical imprecision inherent to the sample size. Therefore, given the limited power of the Hosmer–Lemeshow test in small samples under quintile partitioning, this optimal fit should be interpreted with caution and confirmed in larger-scale external cohorts.

## 3. Discussion

The *CYP2C9* genotype profiles and allele frequency distributions observed in this study are broadly consistent with the genetic architecture described for western Venezuela. The similarity in the prevalence of the CYP2C9*1/*2 genotype to that reported in Lara State suggests a degree of population-level homogeneity for this variant. However, the identification of the homozygous CYP2C9*2/*2 genotype and the differences observed in the frequency of CYP2C9*1/*3 may indicate local genetic heterogeneity influenced by the population’s historical admixture. This variability becomes more evident when compared with the findings of Flores-Gutiérrez et al., in which populations from the same national territory, such as San Antonio and Macanilla in Apure State, exhibited markedly different genotype profiles [36]. These microevolutionary differences may reflect the effects of genetic drift and asymmetric ancestry flow within Venezuelan populations, highlighting the fact that local pharmacogenetic frequencies should not necessarily be extrapolated to the national population.

Comparison with the broader Latin American context revealed marked regional and inter-ethnic variation. Among populations with epilepsy, Peruvian patients showed substantially lower frequencies of CYP2C9*1/*2 (5.62%) and CYP2C9*2/*3 (1.12%) than those observed in the present study, whereas the frequency of CYP2C9*1/*3 was similar to that observed in the Andean population of Mérida, with a difference of 1.32% [37]. In general-population studies, the prevalence of CYP2C9*1/*2 in our cohort was higher than that reported for Mérida, and within the ranges previously reported for Puerto Rico, Colombia, Mexico, the Dominican Republic, and Brazil (10.4–13.2%) while differing from previously reported frequencies in Peru (4.7–5.96%) [20,38]. For CYP2C9*1/*3, frequencies reported in Colombia, Brazil, and Puerto Rico (0.66–3.66%) were higher than those reported in Mexico and the Dominican Republic, whereas Peru showed a wider range (2.4–9.17%) [20,38]. Rare genotypes further illustrated this geographical variation: CYP2C9*2/*2 was reported in Puerto Rico, Brazil, and the Dominican Republic (1.6–1.9%) but was absent from the other countries considered. Similarly, CYP2C9*2/*3 frequencies ranging from 1.0% to 2.1% were reported in Colombia, Brazil, Puerto Rico, and the Dominican Republic, whereas this diplotype was not reported in Mexico and Peru, except for a single case in Peru (0.46%) [20].

The CYP2C9*3-defining locus was in Hardy–Weinberg equilibrium (HWE), indicating that the observed genotype distribution was consistent with that expected under the HWE model. However, this finding should not be interpreted as evidence for the absence of evolutionary forces, selection pressures, or systematic sampling bias, particularly because the study population consisted exclusively of patients with epilepsy. In contrast, the CYP2C9*2-defining locus showed a statistically significant deviation from HWE. Given the clinical nature of the cohort, several biological and methodological factors may potentially contribute to this deviation [39].

Firstly, there is a probable biological association between CYP2C9*2 and the pathophysiology of epilepsy or the therapeutic response to ASMs. ADRs or clinical inefficacy linked to specific CYP2C9*2 genotypes could induce a selection bias through selective survival, altering phenotypic representativeness in the clinical setting. Secondly, the intrinsic limitations of the sample size (*n* = 72) restrict the statistical power to distinguish sampling error from microevolutionary phenomena such as genetic drift or population stratification, where processes like inbreeding increase homozygosity in a real way [40]. Finally, technical artefacts cannot be ruled out, documented as the most frequent methodological cause of deviation from Hardy–Weinberg equilibrium (HWE) in observational studies. Errors in the specificity of molecular primers or probes can induce allelic dropout, prevent the precise discrimination of heterozygotes, and bias the genotypic distribution towards homozygosity.

To validate the fidelity of the genotypic data and rule out that the elevated odds ratios estimated were the product of a technical artefact, a strict quality control protocol was implemented. The automated analysis recorded a call rate of 100% for all polymorphisms and perfect inter-assay concordance (Kappa index of 1.00). Likewise, to resolve the suspicion of possible allelic dropout, the five samples identified with the homozygous variant genotype *2/*2 were validated by Sanger sequencing, which confirmed homozygosity in 100% of cases. Consequently, the molecular findings faithfully reflect the pharmacogenetic architecture of the cohort, ensuring the internal validity of the clinical associations obtained using Firth’s multivariate model.

From a structural perspective, the p.Arg144Cys (CYP2C9*2) and *p.Ile359Leu* (CYP2C9*3) substitutions can alter the structure and functional properties of CYP2C9, potentially affecting the hydrophobicity and electrostatic environment of its active site. These changes may reduce the structural complementarity between the enzyme and its substrates, thereby impairing the metabolism of certain ASMs [41]. According to the Activity Score system of the CPIC [42], the present study identified a prevalence of 26.38% for IM and 4.17% for PM. These findings are clinically relevant because reduced CYP2C9 activity may increase exposure to CYP2C9 substrates, and depending on the medication and therapeutic context, may increase the risk of ADRs.

When comparing our findings with those reported for other Venezuelan populations, the frequency of IMs was similar to that reported in San Antonio (28.5%) and Lara (24.4%) but differed from the 8.6% reported in Macanilla. Notably, the prevalence of PM in the Mérida cohort was higher than that reported in San Antonio (2.8%) and was not observed in the other Venezuelan populations evaluated [36]. A similar pattern was observed when our findings were compared with those reported for a Peruvian mestizo population. The frequency of IMs in the present study, based on the CYP2C9*1/*2 and CYP2C9*1/*3 diplotypes, was 8.2 percentage points higher, whereas the frequency of PMs exceeded that previously reported for the Peruvian population by 3.05 percentage points [37].

The findings of this study indicate that variability in the safety profile of ASM monotherapy was more strongly associated with CYP2C9 metaboliser phenotype than with the specific ASM prescribed. No statistically significant association was observed between the type of ASM prescribed as monotherapy and the occurrence of ADRs (*p* = 0.74), whereas a statistically significant association was observed between CYP2C9 metaboliser phenotype and ADR occurrence (*p* < 0.001). The high proportion of patients experiencing ADRs among IMs (84.2%) and PMs (100%) is consistent with the established pharmacological consequences of reduced CYP2C9 activity. Reduced CYP2C9 activity can decrease the clearance of CYP2C9 substrates with a narrow therapeutic index, such as PHT, thereby increasing systemic exposure and potentially increasing the risk of dose-dependent toxicity [13]. However, the magnitude of this effect depends on the specific drug, its dependence on CYP2C9-mediated metabolism, and other pharmacokinetic and clinical factors.

The relatively low overall incidence of ADRs observed in some subgroups receiving monotherapy may also reflect the pharmacokinetic properties of the individual ASMs. CBZ, for example, is a well-established inducer of several drug-metabolising enzymes, including CYP3A4, CYP2C9, CYP2C19, UGT1A4, and microsomal epoxide hydrolase [43,44]. Through enzyme induction, CBZ can increase the metabolism of co-administered drugs that are substrates of these pathways, including LTG, which is primarily eliminated by glucuronidation [43,45]. Such interactions may reduce the plasma concentrations of some co-administered ASMs, and depending on the clinical context, may affect both treatment efficacy and safety. Therefore, the absence of ADRs in some patients should not necessarily be interpreted as evidence of improved drug clearance attributable to their *CYP2C9* genotype, particularly in the absence of therapeutic drug monitoring or pharmacokinetic measurements.

In contrast, the present study observed a higher proportion of ADRs among patients with the CYP2C9*1/*1 genotype receiving polytherapy than among patients classified as IMs or PMs. This finding differs from the expected association between reduced CYP2C9 activity and increased exposure to CYP2C9 substrates and should be interpreted cautiously. Several factors may account for this observation, most importantly, the very small size of the polytherapy subgroup (*n* = 7), in which each patient represented 14.29% of the subgroup, as well as the heterogeneity of the treatment regimens administered.

The composition of the polytherapy regimens provides a possible pharmacological explanation for this pattern. VPA can inhibit the metabolism of several concomitant ASMs and is particularly relevant to the pharmacokinetics of LTG. VPA inhibits LTG glucuronidation and can substantially increase LTG exposure, thereby potentially increasing the risk of adverse reactions [43,44,45]. Consequently, in patients receiving VPA-containing combinations, the pharmacokinetic effects of drug–drug interactions may have contributed substantially to the occurrence of ADRs, independently of CYP2C9 metaboliser phenotype. This possibility is particularly relevant because several patients in the polytherapy subgroup received VPA in combination with LTG, with or without LEV.

Furthermore, interpretation of the *CYP2C9* genotype in this subgroup is limited by the pharmacological characteristics of the drugs administered. LEV undergoes extensive enzymatic hydrolysis outside the hepatic CYP450 system, whereas LTG is predominantly eliminated through glucuronidation. Thus, these drugs are not expected to exhibit the same degree of dependence on CYP2C9-mediated metabolism as PHT or other CYP2C9 substrates. Accordingly, the observed ADRs among NM receiving polytherapy may reflect the combined effects of treatment regimen, drug–drug interactions, and individual susceptibility rather than an intrinsic increase in ADR risk attributable to the *CYP2C9* genotype.

These findings should nevertheless be regarded as preliminary. The small sample size, particularly within the polytherapy subgroup, the heterogeneity of treatment combinations, and the absence of direct pharmacokinetic measurements limit the extent to which mechanistic conclusions can be drawn. Larger, multicentre studies incorporating detailed treatment histories, therapeutic drug monitoring, and systematic pharmacovigilance are warranted to determine the independent contributions of *CYP2C9* genotype, ASM exposure, and drug–drug interactions to ADR risk in this population.

In line with the previous literature, the occurrence of ADRs varies according to the specific ASM, dosage, treatment regimen, and individual patient characteristics [46]. Commonly reported adverse effects include sedation, asthenia, dizziness, ataxia, dysarthria, diplopia, tremor, cognitive impairment, mood disturbances, and sexual dysfunction [47]. Gastrointestinal symptoms, including nausea, vomiting, and abdominal pain, have also been reported [48]. Furthermore, LEV, topiramate, zonisamide, vigabatrin, and perampanel have been associated with psychiatric and behavioural adverse effects, including anxiety, depression, irritability, emotional lability, hyperactivity, attention disturbances, and in rare cases, psychosis [49].

VPA, in turn, has been associated with weight gain, hand tremor [7,50], hepatotoxicity including severe liver injury and liver failure [51], and hyperammonaemic encephalopathy, particularly in individuals with underlying metabolic disorders such as ornithine transcarbamylase deficiency [52]. Other ASMs, including CBZ, gabapentin, pregabalin, perampanel, and vigabatrin, have also been associated with weight gain, which may contribute to an increased risk of obesity and cardiovascular disease [50,53]. Conversely, LTG has been associated with an increased risk of ventricular arrhythmias and cardiac arrest in patients with underlying cardiac disease [54,55], whereas topiramate, zonisamide, and felbamate are generally associated with weight loss [49].

Among the enzyme-inducing ASMs, PHT is associated with adverse effects such as hirsutism and gingival hyperplasia, whereas PHB may cause drowsiness and sedation and has been associated with shoulder–hand syndrome and Dupuytren’s contracture [50]. Furthermore, CBZ and OXC may exacerbate seizures in specific epilepsy syndromes, particularly in patients with juvenile myoclonic epilepsy and other generalised epilepsies in which sodium-channel blockade may aggravate seizure activity [56].

The occurrence of these ADRs may be partly explained by the relatively narrow therapeutic indices of some of these ASMs, for which relatively small increases in systemic exposure may result in plasma concentrations approaching or exceeding the therapeutic range, and in some circumstances, increase the risk of toxicity [6,7]. Furthermore, CBZ, PHT, and VPA exhibit substantial plasma protein binding, with approximately 76%, 90%, and 90% bound to albumin, respectively [57]. In this context, malnutrition and the resulting hypoalbuminaemia may increase the unbound or pharmacologically active fraction of highly protein-bound drugs, potentially increasing drug exposure at the site of action and contributing to toxicity and ADRs [58].

In patients receiving polytherapy for epilepsy, the potential for drug–drug interactions should also be considered. VPA can competitively displace PHT from plasma protein-binding sites, increasing the unbound fraction of PHT. This interaction may contribute to changes in the relationship between total and free PHT concentrations and may increase the risk of toxicity, particularly when the total plasma concentrations are interpreted without considering protein binding and other pharmacokinetic factors [43].

Although this study represents an initial contribution to the characterisation of *CYP2C9* allelic variation and its potential association with ADRs in patients with epilepsy from the Andean region of Mérida, Venezuela, its observational design and the genetic heterogeneity of the Venezuelan population require that the findings be interpreted with caution. Nevertheless, the results support the need for further research aimed at evaluating the potential role of clinical pharmacokinetics and personalised medicine approaches in optimising the safety of antiseizure medication therapy in this population.

The findings of this study should also be interpreted in light of several methodological limitations. First, the genetic analysis was restricted to the CYP2C9*2 and CYP2C9*3 alleles and therefore did not capture other potentially relevant pharmacogenetic variants. In particular, polymorphisms in genes encoding other drug-metabolising enzymes, such as CYP2C19, CYP3A4, and CYP3A5, were not evaluated. Furthermore, variants in genes encoding drug efflux transporters, including *ABCB1* and *ABCC2*, were not characterised. Consequently, the potential contribution of transporters such as P-glycoprotein (P-gp) and multidrug resistance-associated protein 2 (MRP2) respectively to interindividual variability in drug disposition and treatment response could not be assessed. The absence of these genetic and pharmacokinetic variables may have resulted in residual confounding and limits the extent to which the observed associations can be attributed specifically to *CYP2C9* variation. Second, the lack of measurement of steady-state plasma concentrations of ASMs precluded a direct assessment of the relationship between *CYP2C9* genotype, predicted metaboliser phenotype, and systemic drug exposure. Therapeutic drug monitoring and longitudinal pharmacokinetic assessment would therefore be valuable in future studies to determine whether the predicted differences in CYP2C9 activity translate into clinically relevant differences in drug concentrations. Third, the absence of a standardised instrument for assessing the causality and severity of ADRs represents an additional methodological limitation. In particular, the use of a validated causality assessment tool, such as the Naranjo algorithm, together with a standardised severity classification system, such as the National Cancer Institute’s Common Terminology Criteria for Adverse Events (NCI-CTCAE), could have improved the consistency and reproducibility of ADR classification. The absence of such standardised assessments may have introduced misclassification bias in the identification and characterisation of ADRs. Finally, the relatively small sample size (*n* = 72) and the clinical heterogeneity of the cohort limited statistical power and the ability to account adequately for potential confounding factors and multiple comparisons. These limitations are particularly relevant given the small number of patients receiving polytherapy and the heterogeneity of ASM combinations. Consequently, the observed associations should be considered exploratory and require confirmation in larger, prospective, multicentre cohorts before their clinical applicability can be established.

Nevertheless, these limitations do not preclude the potential clinical relevance of the present findings. To our knowledge, this study represents the first pharmacogenetic investigation of patients with epilepsy from the Andean region of Mérida, Venezuela, and provides preliminary data on *CYP2C9* genetic variation and its potential association with ASM-related ADRs in this population. These findings contribute to addressing the limited representation of this population in the pharmacogenetic literature and provide a basis for the further investigation of precision medicine approaches in the region.

Furthermore, the present study provides a foundation for subsequent prospective, multicentre investigations, currently under consideration, aimed at characterising the relationship between *CYP2C9* genetic variation, predicted metaboliser phenotype, plasma drug concentrations, and susceptibility to ADRs. Such studies should incorporate longitudinal pharmacokinetic monitoring, standardised assessment of ADR causality and severity, and larger and more representative patient cohorts to determine the clinical relevance of CYP2C9-guided optimisation of ASM therapy.

## 4. Materials and Methods

### 4.1. Study Design and Setting

A cross-sectional observational study was conducted between January and December 2025 at the Neurology Teaching and Clinical Care Unit of the Autonomous Institute of the University Hospital of the Andes (IAHULA), Faculty of Medicine, Mérida, Venezuela.

### 4.2. Study Population and Sampling

Participants were recruited using a non-probability convenience sampling strategy with consecutive enrolment. Eligible participants were adults diagnosed with focal epilepsy, generalised epilepsy, or combined focal and generalised epilepsy according to established clinical and electroencephalographic criteria.

Following application of the predefined inclusion and exclusion criteria, the final study population comprised 72 patients. The cohort included 44 women (61.11%; age range, 20–94 years) and 28 men (38.89%; age range, 18–88 years).

### 4.3. Eligibility Criteria

Patients attending the Neurology Teaching and Clinical Care Unit of the Autonomous Institute of the University Hospital of the Andes (IAHULA) were invited to participate in the study. Following their routine neurological evaluation, eligible patients received detailed information regarding the objectives, procedures, and significance of the study.

A total of 100 patients were screened, of whom 72 met the eligibility criteria and were enrolled. The inclusion criteria were as follows: (i) a diagnosis of epilepsy; (ii) treatment with ASMs for at least one year, either as monotherapy (CBZ, VPA, PHT, PHB, or OXC) or polytherapy (VPA + LTG, VPA + LEV, PHB + CBZ + LTG, CBZ + LEV, or VPA + LTG + LEV); (iii) controlled or uncontrolled epilepsy, including patients classified as poor responders to polytherapy; (iv) adherence to the prescribed treatment, defined as taking the medication at the scheduled times with approximately 250 mL of water; (v) agreement to avoid self-medication and to report the use of any additional medications; (vi) birth in Mérida State, Venezuela; (vii) age ≥ 18 years; (viii) willingness to provide a 5-mL peripheral blood sample for *CYP2C9* SNP analysis; and (ix) provision of written informed consent before enrolment.

Twenty-eight patients who did not meet the eligibility criteria were excluded, mainly due to pregnancy status, the presence of severe comorbidities (severe hepatic or renal insufficiency), or concomitant treatments that interfere with the metabolism of the assessed ASMs.

#### Patient Flow and Breakdown

Initially, 100 patients with epilepsy were assessed (Table 7). Of these, 16 were excluded during the baseline phase for not meeting the eligibility criteria: 10 due to low initial adherence and 6 due to being pregnant. The remaining 84 patients were formally included after signing the informed consent form. During the follow-up phase, 12 losses were recorded due to voluntary withdrawal or loss of contact. Finally, the analysed sample consisted of 72 patients who had complete clinical data and successful genotyping of the *CYP2C9* gene [59].

### 4.4. Genomic DNA Extraction

Peripheral venous blood samples were collected by venipuncture. Genomic DNA (gDNA) was extracted from the leukocyte fraction using the DNeasy Blood & Tissue^®^ Kit (QIAGEN, Hilden, Germany), according to the manufacturer’s instructions. DNA concentration and purity were assessed spectrophotometrically using a DeNovix DS-11 FX Series Spectrophotometer™ (DeNovix Inc., Wilmington, DE, USA). DNA samples were considered suitable for downstream analyses when the A260/A280 and A260/A230 absorbance ratios were ≥1.7. Purified gDNA samples were stored at −20 °C until genotyping.

### 4.5. CYP2C9 Genotyping

The CYP2C9*2 (rs1799853; c.430C>T) and CYP2C9*3 (rs1057910; c.1075A>C) allelic variants were determined by real-time polymerase chain reaction (qPCR) using TaqMan^®^ SNP Genotyping Assays (Thermo Fisher Scientific, Waltham, MA, USA). Amplification reactions were performed in a final volume of 10 µL, comprising 5 µL of TaqMan^®^ Genotyping Master Mix (cat. no. 4371355), 0.5 µL of the corresponding probe and primer mixture (assays C_25625805_10 and C_27104892_10), 20 ng of genomic DNA (2 µL at 10 ng/µL), and molecular biology-grade nuclease-free water (HyPure, HyClone, Logan, UT, USA).

All genotyping assays were performed in duplicate. The context sequences corresponding to each assay are provided in Table 8.

Genotype assignment and allelic discrimination were performed based on the endpoint fluorescence signals generated by the FAM and VIC fluorophores. Strict quality-control procedures were implemented for each assay. These included a No-Template Control (NTC), consisting of molecular biology-grade nuclease-free water, to monitor potential contamination, and three previously characterised positive controls for each SNP. The positive controls represented the homozygous reference, heterozygous, and homozygous alternative genotypes: C/C, C/T, and T/T for rs1799853, and A/A, A/C, and C/C for rs1057910. The thermal cycling programme was performed using a Stratagene Mx3000P™ real-time PCR system (Agilent Technologies, Waldbronn, Germany) under the following conditions: initial enzyme activation at 95 °C for 10 min, followed by 40 cycles of denaturation at 95 °C for 15 s, and a combined annealing and extension step at 60 °C for 90 s.

### 4.6. Assessment of ADRs

ADRs were identified during routine neurological follow-up through systematic clinical interviews and a comprehensive review of medical records. The adverse events assessed included distal upper-limb tremor, weight gain, drowsiness, vertigo, and tongue paraesthesia. An event was operationally classified as an ADR when it was clinically relevant and documented by the treating neurologist during exposure to one or more ASMs.

For the statistical analysis, patients were categorised according to the presence or absence of at least one documented ADR. Consistent with the real-world observational design of the study, causality was assessed based on the treating specialist’s clinical judgement and the temporal relationship between ASM exposure and the reported event, without the use of a formal algorithmic causality assessment tool such as the Naranjo algorithm. Similarly, ADR severity was assessed according to the clinical impact documented in the medical records rather than by applying a standardised semi-quantitative classification system such as the National Cancer Institute’s Common Terminology Criteria for Adverse Events (NCI-CTCAE).

### 4.7. Statistical Analysis

Categorical variables were compared using Fisher’s exact test to analyse the association between epilepsy type and sex, as well as to assess sex differences in pharmacotherapy with valproic acid. Using the Mann–Whitney U test, differences associated with sex were assessed.

The selection of the independent variables was initially based on expert judgement, tentatively including *CYP2C9* metabolic phenotype, ASM polytherapy, type of epilepsy, sex, and age. Nonetheless, in order to prioritise parsimony of the model and prevent overfitting due to the reduced sample size, only those variables that modified the effect or demonstrated direct clinical relevance were retained in the final model, with age and sex being excluded from the definitive multivariate analysis.

The diagnosis of the model included the assessment of multicollinearity using the variance inflation factor (VIF), ruling out relevant interactions. In addition, the calibration of the model was evaluated with the Hosmer–Lemeshow goodness-of-fit test. The 95% confidence intervals (95% CIs) for the odds ratios (ORs) were calculated using the likelihood profile, and the *p*-values were derived from the penalised likelihood ratio test. The estimates were considered statistically significant if *p* < 0.05.

Statistical analyses were performed in Python (version 3.9; Python Software Foundation, Wilmington, DE, USA), using the pandas libraries for data management and statsmodels for modelling and validation of the results.

### 4.8. Ethical Considerations

This study was conducted in collaboration with the Neurology Teaching and Clinical Care Unit of the Faculty of Medicine, Autonomous Institute of the University Hospital of the Andes (IAHULA), Mérida, Venezuela. The study protocol and informed consent form were reviewed and approved by the Neurology Committee of IAHULA as a minimal-risk study involving peripheral blood samples collected during routine clinical care (Approval No. 001-SN-IAHULA-2025, 18 February 2025).

The study was conducted in accordance with the ethical principles of the Belmont Report and the Declaration of Helsinki, including its applicable revisions. All participants provided written informed consent before enrolment.

To protect participant anonymity and ensure data confidentiality, the following measures were implemented: (i) each participant was assigned a unique study code for the collection and analysis of clinical and research data; (ii) access to clinical records, pharmacological treatment information, and pharmacogenetic results was restricted to authorised investigators; and (iii) the informed consent document described the objectives and significance of the study, the intended use of the data for scientific publication, the confidentiality safeguards in place, and the participants’ right to withdraw from the study at any time without penalty.

## 5. Conclusions

The present study identified the CYP2C9*2 and CYP2C9*3 allelic variants in patients with epilepsy from the Venezuelan Andean region. These variants contributed to the observed distribution of CYP2C9 metaboliser phenotypes, including intermediate and PM, and CYP2C9 metaboliser status was significantly associated with the occurrence of ADRs in patients receiving ASMs.

The observed association between CYP2C9 metaboliser phenotype and ADR occurrence supports the potential clinical relevance of pharmacogenetic assessment in the management of patients receiving ASMs. *CYP2C9* genotyping may provide additional information for identifying patients with reduced CYP2C9 activity who could be at increased risk of adverse effects from susceptible CYP2C9 substrates. However, given the observational design, relatively small sample size, and absence of pharmacokinetic measurements in the present study, these findings should be considered preliminary and should not be interpreted as establishing a basis for genotype-guided dose adjustment in this population.

Further clinical and pharmacogenetic research is warranted in larger, prospective, and more diverse populations from Mérida and other regions of Venezuela and Latin America. Future studies should incorporate therapeutic drug monitoring and longitudinal pharmacokinetic assessment to determine whether CYP2C9 metaboliser phenotypes are associated with clinically relevant differences in plasma drug concentrations and whether these differences contribute to the occurrence and severity of ASM-related ADRs. Such investigations may provide the evidence required to determine the clinical utility of CYP2C9-guided approaches for optimising the safety and effectiveness of antiseizure therapy.

## Figures and Tables

**Figure 1:**
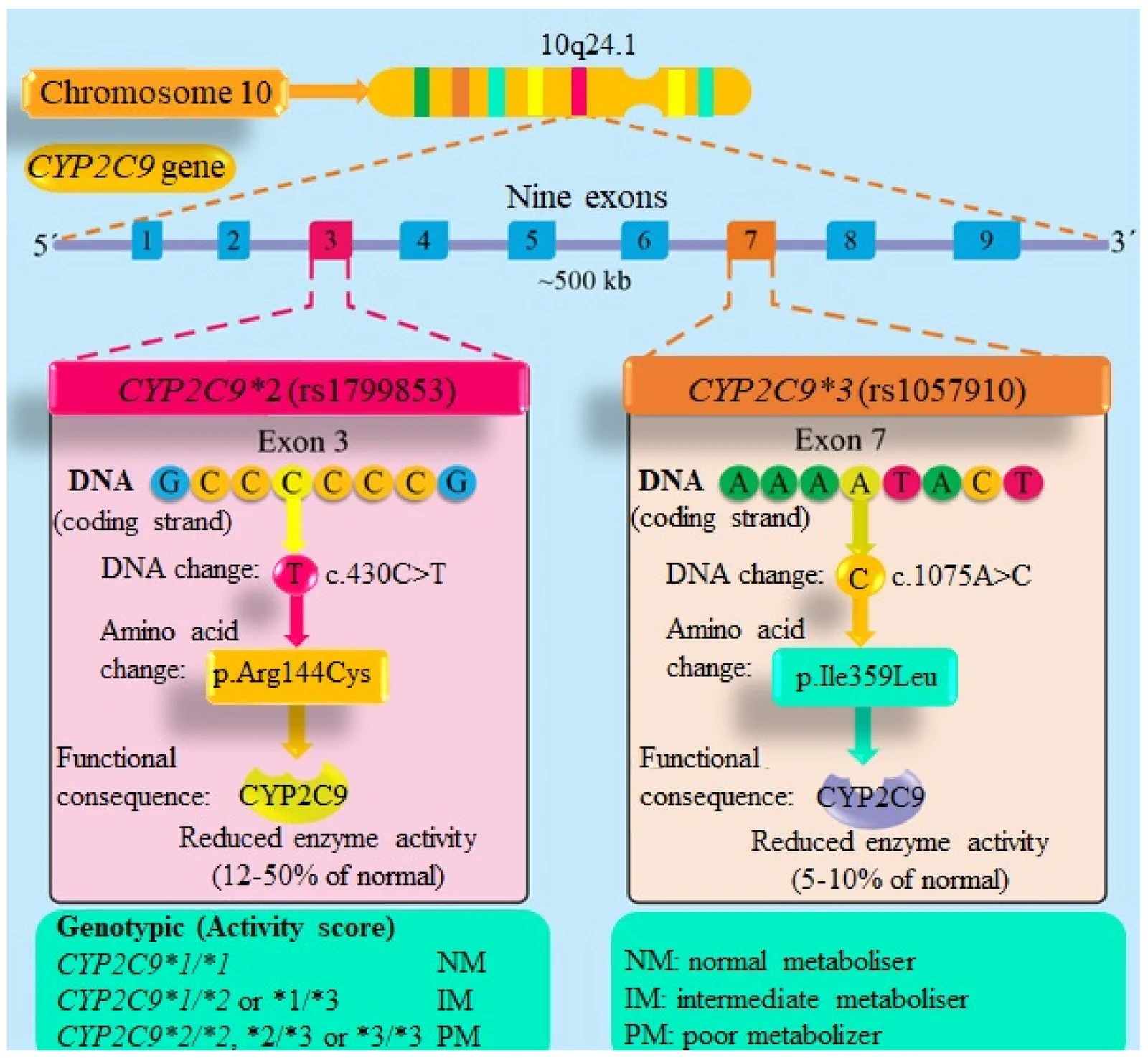
Genomic location and functional consequences of the CYP2C9*2 (rs1799853) and CYP2C9*3 (rs1057910) variants. Schematic representation of the *CYP2C9* gene on chromosome 10q24.1 showing the location of the CYP2C9*2 (c.430C>T; p. Arg144Cys) and CYP2C9*3 (c.1075A>C; p. Ile359Leu) variants in exons 3 and 7, respectively. Both variants encode enzymes with reduced metabolic activity and are associated with intermediate or poor metaboliser phenotypes depending on the allelic combination.

**Table 1:** Demographic and clinical characteristics of patients.

Variable	Female (*n* = 44, 61.11%)			Male (*n* = 28, 38.89%)			*p*-Value
	Mean ± SD		Range	Mean ± SD		Range	
Age (years)	46.45 ± 17.54		20–94	43.21 ± 18.85		18–88	0.429 *
Weight (kg)	60.79 ± 11.46		40–97	66.01± 14.46		39–97.50	0.066 *
Monotherapy	*n* = 40 (55.55%)	dose (mg/day)		*n* = 25 (34.72%)	dose (mg/day)		1.00 **
CBZ (Mean ± SD)	16 (22.22%)	650 ± 89.44	600–800	4 (5.55%)	650 ± 100	600–800	
VPA (Mean ± SD)	13 (18.05%)	1730.77 ± 259.44	1500–2000	16 (22.22%)	1593.75 ± 201.56	1500–2000	
PHT	10 (13.89%)	300	300	2 (2.78%)	300	300	
PHB	1 (1.39%)	300	300	2 (2.78%)	300	300	
OXC	0 (0.0)			1 (1.39%)	600	600	
Polytherapy	*n* = 4 (5.56%)	dose (mg/day)		*n* = 3 (4.17%)	dose (mg/day)		
VPA LTG	1 (1.39%)	1500 100	1500 100	1 (1.39%)	1500 100	1500 100	
VPA LEV	1 (1.39%)	1000 3000	1000 3000	1 (1.39%)	1000 3000	1000 3000	
PHB CBZ LTG	1 (1.39%)	300 100 100	300 100 100	0 (0.0)			
CBZ LEV	1 (1.39%)	300 3000	300 3000	0 (0.0)			
VPA LTG LEV	0 (0.0)			1 (1.39%)	1000 100 3000	1000 100 3000	
Epilepsy type							
Generalised	23 (52.27%)			13 (46.43%)			0.848 **
Focal	6 (13.64%)			5 (17.86%)			
Combined	15 (34.09%)			10 (35.71%)			
Controlled (without recurrence)	13 (29.55%)			11 (39.29%)			0.550 **
Not controlled	31 (70.45%)			17 (60.71%)			

CBZ: carbamazepine; VPA: valproic acid; PHT: phenytoin; PHB: phenobarbital; OXC: oxcarbazepine; LTG: lamotrigine; LEV: levetiracetam; SD: standard deviation; Combined (generalised and focal). * *p*-values were calculated using the Mann–Whitney U test for continuous variables. ** Categorical variables were compared using Pearson’s chi-square test.

**Table 2:** Marginal genotype frequencies of CYP2C9*2 and CYP2C9*3 in the study population of patients.

Allele	*n*	f	Genotype	*n* (%)
*1	122	0.85	*1/*1	55 (76.39)
*2	22	0.15	*1/*2	12 (16.67)
			*2/*2	5 (6.94)
Total	144	1.00		72 (100%)
*1	136	0.94	*1/*1	64 (89.00)
*3	8	0.06	*1/*3	08 (11.00)
			*3/*3	0 (0.00)
Total	144	1.00		72 (100.00%)

Genotype frequencies are presented separately for each *CYP2C9* locus. rs1799853 (c.430C>T) defines the CYP2C9*2 allele, whereas rs1057910 (c.1075A>C) defines the CYP2C9*3 allele.

**Table 3:** Inferred *CYP2C9* diplotype frequencies and predicted metaboliser phenotypes.

Genotypes	*n (%)*	Activity Score	Metaboliser Phenotype (%)
*1/*1	50 (69.45)	2.0	NM (69.45)
*1/*2	9 (12.50)	1.5	IM (26.38)
*1/*3	5 (6.94)	1.0	
*2/*2	5 (6.94)	1.0	
*2/*3	3 (4.17)	0.5	PM (4.17)
*3/*3	0 (0.00)	0.0	
total	72 (100%)		

Predicted CYP2C9 metaboliser phenotypes were assigned according to CPIC activity score thresholds: normal metaboliser (NM) AS = 2, intermediate metaboliser (IM) AS = 1 or 1.5, and poor metaboliser (PM) AS = 0 or 0.5 [13].

**Table 4:** CYP2C9 Metaboliser phenotypes and adverse drug reactions associated with antiseizure medication monotherapy.

Phenotype/ Genotype	ASM	ADR *n* (%)	No ADR *n* (%)	Total *n* (%)
Normal metaboliser CYP2C9*1/*1	CBZ	3 (4.62)	12 (18.46)	15 (23.08)
	OXC	0 (0.00)	1 (1.54)	1 (1.54)
	PHB	2 (3.08)	1 (1.54)	3 (4.62)
	PHT	2 (3.08)	8 (12.31)	10 (15.38)
	VPA	2 (3.08)	12 (18.46)	14 (21.54)
Intermediate metaboliser CYP2C9*1/*2	CBZ	2 (3.08)	0 (0.00)	2 (3.08)
	VPA	5 (7.69)	2 (3.08)	7 (10.77)
Intermediate metaboliser CYP2C9*1/*3	CBZ	1 (1.54)	0 (0.00)	1 (1.54)
	PHT	1 (1.54)	0 (0.00)	1 (1.54)
	VPA	2 (3.08)	1 (1.54)	3 (4.62)
Intermediate metaboliser CYP2C9*2/*2	CBZ	1 (1.54)	0 (0.00)	1 (1.54)
	PHT	1 (1.54)	0 (0.00)	1 (1.54)
	VPA	3 (4.62)	0 (0.00)	3 (4.62)
Poor metaboliser CYP2C9*2/*3	CBZ	1 (1.54)	0 (0.00)	1 (1.54)
	VPA	2 (3.08)	0 (0.00)	2 (3.08)
Total:		28 (43.08)	37 (56.92)	65 (100.00)

ASM: antiseizure medication; ADR: adverse drug reaction; No ADRs: No adverse drug reactions. CBZ: carbamazepine; VPA: valproic acid; PHT: phenytoin; PHB: phenobarbital; OXC: oxcarbazepine; LTG: lamotrigine; Chi-square test for the presence of ADRs according to *CYP2C9* phenotype (*p* = 0.001). Chi-square test for the presence of ADRs according to ASM in monotherapy (*p* = 0.741).

**Table 5:** Distribution of adverse drug reactions according to the polytherapy regimen with antiseizure medications and the *CYP2C9* genotype.

Polytherapy Regimen (ASM)	CYP1C9*1/*1 Genotype (NM) with ADR/ Without ADR (*n*)	Other Genotypes with ADRs/ Without ADRs (*n*)	Patients by Regimen (*n*)	Total ADR Incidence by Regimen (%)
VPA + LTG	2/0	0/0	2	100.0 (2/2)
VPA+ LEV	1/1	0/0	2	50.0 (1/2)
VEPA + LTG + LEV	1/0	0/0	1	100.0 (1/1)
PHB + CBZ + LTG	1/0	0/0	1	100.0 (1/1)
CBZ + LEV	1/0	0/0	1	100.0 (1/1)
Total	6/1	0/0	7	85.71% (6/7)

ASM: antiseizure medication; ADR: adverse drug reaction; No ADRs: No adverse drug reactions. CBZ: carbamazepine; VPA: valproic acid; PHB: phenobarbital; LTG: lamotrigine; LEV: levetiracetam.

**Table 6:** Firth’s penalised logistic regression analysis of factors associated with adverse reactions to antiseizure medications.

Predictor	Reference	OR	95%CI	*p*-Value
IM or PM	NM	17.45	5.09–76.56	<0.001
Polytherapy	Monotherapy	15.03	2.73–157.37	0.001
Generalised epilepsy	Focal	1.43	0.28–8.29	0.672
Combined epilepsy	Focal	0.85	0.15–5.26	0.857

CI: confidence interval; IM: intermediate metaboliser; NM: normal metaboliser; OR: odds ratio; PM: poor metaboliser. The ORs and 95% CIs were estimated using Firth’s penalised logistic regression to account for the small sample size and censoring. *p*-values were derived from the penalised likelihood ratio test. Hosmer–Lemeshow chi-squared goodness-of-fit χ^2^ = 0.67 (df = 3, *p* = 0.880), based on five risk groups due to tied predicted probabilities. Maximum variance inflation factor (VIF) = 2.19.

**Table 7:** Participant flow and selection criteria throughout the study phases.

Protocol Phase	Patients (*n*)	Details and Criteria Applied
Initially evaluated	100	Patients with epilepsy recruited in the Neurology Unit (IAHULA).
Excluded in basal phase	16	Not eligible: pregnancy status (*n* = 6) or low baseline adherence (*n* = 10).
Formally included	84	Subjects who met the selection criteria and gave informed consent.
Losses in follow-up	12	Voluntary withdrawal of consent or loss of post-inclusion contact.
Final sample analysed	72	Patients with complete clinical records and successful genotyping of *CYP2C9*.

**Table 8:** TaqMan^®^ assay context sequences for the *CYP2C9* single-nucleotide polymorphisms (SNPs) investigated in this study.

Allele (dbSNP)	Context Sequences	TaqMan^®^ Assay
CYP2C9*2 (rs1799853)	GATGGGGAAGAGGACATTGAGGAC[**C/T**] GTGTTCAAGAGGAAGCCCGCTGCCT	C__25625805_10
CYP2C9*3 (rs1057910)	TGTGGTGCACGAGGTCCAGAGATAC[**C/A**] TTGACCTTCTCCCCACCAGCCTGCC	C__27104892_10

dbSNP: Database of Single Nucleotide Polymorphisms. CYP2C9*2/*3 genotype was inferred from the combination of rs1799853 and rs1057910 genotypes, based on the established haplotype structure of *CYP2C9*, in which the variants defining alleles *2 and *3 are rarely observed in cis [60].

## Data Availability

The original contributions presented in this study are included in the article. Further inquiries can be directed to the corresponding author.

## Abbreviations

The following abbreviations are used in this manuscript:

ADRs	Adverse drug reactions
ASMs	Antiseizure medications
CPIC	Clinical Pharmacogenetics Implementation Consortium
gDNA	Genomic DNA
IAHULA	Autonomous Institute of the University Hospital of the Andes
95% CI	95% confidence interval
OR	Odds ratio
IM	Intermediate metabolisers
NM	Normal metabolisers
PM	Poor metabolisers
CBZ	Carbamazepine
LEV	Levetiracetam
LTG	Lamotrigine
OXC	Oxcarbazepine
PHT	Phenytoin
PHB	Phenobarbital
VPA	Valproic acid
SNP	Single nucleotide polymorphism
